# Ureteroscopy for Stone Disease in Paediatric Population is Safe and Effective in Medium-Volume and High-Volume Centres: Evidence from a Systematic Review

**DOI:** 10.1007/s11934-017-0742-3

**Published:** 2017-10-18

**Authors:** Shazna Rob, Patrick Jones, Amelia Pietropaolo, Stephen Griffin, Bhaskar K. Somani

**Affiliations:** 1 Department of Urology, University Hospital Southampton, Southampton, UK; 2 Department of Paediatric Urology, University Hospital Southampton, Southampton, UK

**Keywords:** Paediatric, Ureteroscopy, Volume, Complications, Success, Urolithiasis

## Abstract

**Purpose of Review:**

The incidence of urinary stone disease among the paediatric population is increasing. Whilst there has been a rise in the number of original studies published on ureteroscopy (URS) in children, critical review still remains under-reported.

**Recent Findings:**

A Cochrane style systematic review was performed to identify all original articles on URS (minimum of 25 cases) for stone disease in paediatric patients between Jan. 1996 and Dec. 2016. Based on the number of reported cases, centres were divided into medium (25–49 cases) and high (≥ 50 cases) volume studies.

Thirty-four studies (2758 children) satisfied our search criteria and were included in this review. The mean stone size was 8.6 mm with an overall stone-free rate (SFR) of 90.4% (range 58–100). Medium-volume centres reported a mean SFR of 94.1% (range 87.5–100), whilst high-volume centres reported a mean SFR of 88.1% (range 58–98.5). Mean number of sessions to achieve stone-free status in medium-volume and high-volume groups was 1.1 and 1.2 procedures/patient respectively. The overall complication rate was 11.1% (327/2994). Breakdown by Clavien grade was as follows: Clavien I 69% and Clavien II/III 31%. There were no Clavien IV/V complications, and no mortality was recorded across any of the studies. The overall failure to access rate was 2.5% (76/2944).

Medium-volume and high-volume studies had overall complication rates of 6.9% (37/530) and 12.1% (287/2222) respectively, but there was no significant difference in major or minor complications between these two groups.

**Summary:**

Ureteroscopy is a safe and effective treatment for paediatric stone disease. Medium-volume centres can achieve equally high SFRs and safety profiles as high-volume centres. Despite the rarity of paediatric stone disease, our findings might increase the uptake of paediatric URS procedures.

## Introduction

The incidence of urinary stone disease among the paediatric population is rising [1]. This has led to the development of minimally invasive and effective endourological interventions that can yield a high stone clearance whilst preserving renal function with low morbidity in these children. In the adult population, application of ureteroscopy (URS) globally has expanded over 200% in the past decade [2•]. This shift owes largely to major advances in surgical technique, laser technology and equipment minimisation. Similar changes have been mirrored in the management of paediatric stone disease, although Ritchey et al. first described URS in a young child in 1988 [3]. Whilst there has been a rise in the number of original studies published on this topic, critical evaluation of the safety and efficacy of URS for paediatric cases remains under-reported. The objective of this study was to therefore formally appraise the existing evidence. Furthermore, given the dissemination of URS and that its uptake is no longer limited to specialist centres, we sought to determine if there were any differences in clinical outcomes among these high-volume centres compared to those reporting medium volumes.

## Material and Methods

### Evidence Acquisition: Criteria for Considering Studies for This Review

#### Inclusion Criteria

- Studies reporting on outcomes following ureteroscopy in paediatric populations
- Patients aged ≤ 18

#### Exclusion Criteria

- Study sample size < 25 patients
- Non-English language articles
- Animal studies

### Search Strategy and Study Selection

**Fig. 1:**
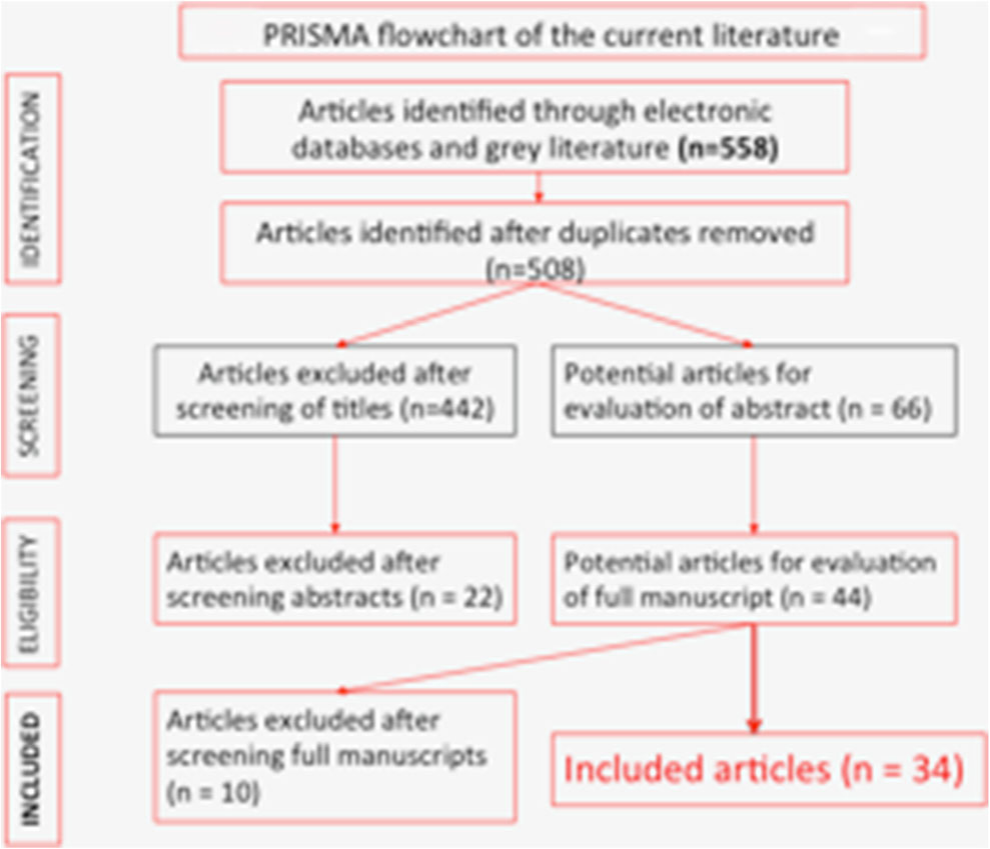
PRISMA flow chart of the current literature

A Cochrane style search was performed to identify all original articles investigating ureteroscopy in paediatric patients (Fig. 1). The Preferred Reporting Items for Systematic Reviews and Meta-Analyses (PRISMA) checklist was adhered to accordingly. Sensitive and customised search strategies were applied to the following online bibliographic databases: PubMed/MEDLINE, EMBASE, CINAHL and The Cochrane Central Register of Controlled Trials, whilst citation lists and study references were also evaluated.

Search terms included (but not limited to) ‘ureteroscopy’, ‘URS’, ‘retrograde intra-renal surgery’, ‘RIRS’, ‘paediatric’, ‘pediatric’, ‘urolithiasis’ and ‘stones’. Boolean operators (AND, OR) were incorporated to refine the search. Medical subject headings (MeSH) included (not limited to) [Urinary calculi], [Ureteroscopy], [Lasers], [Child] and [Nephrolithiasis].

All study types were considered for potential inclusion. A time restriction was applied to include relevant studies published between January 1990 and December 2016. Paediatric age was defined as 18 years or less. Studies combining adult and paediatric populations with no breakdown of results were excluded.

### Outcomes of Interest

#### Primary Outcomes

- Procedure-related complications (graded according to Clavien-Dindo system)
- Stone-free rates (SFRs)

#### Secondary Outcomes

- Comparison of outcomes for ureteroscopy performed in medium-volume centres (reporting 25–49 procedures) and high-volume centres (reporting ≥ 50 procedures).

## Data Extraction and Analysis

Both the search process and data extraction were performed by two authors (SR, PJ) independently and overseen by the senior author (BKS). Information was also collected on patient characteristics, total number of procedures performed and stone location. For the purposes of this review, centres reporting on 25–49 procedures were termed ‘medium-volume’ and ≥ 50 procedures as ‘high-volume’ centres. We did not include studies from centres that reported on < 25 procedures, which were deemed to be low-volume centres. Complications recorded intra-operatively or within the study follow period were included for analysis.

Chi-squared test and independent *t* test were used for dichotomous and continuous data respectively (SPSS version 21).

## Results

Thirty-four studies [4–24, 25•, 26–32, 33••, 34–37] satisfied our search criteria and were included in this review (Table 1). These were all published between 1996 and 2016. A total of 2758 children underwent URS for urinary stone disease. The mean age was 7.8 years (range 0.25–18) with a male to female ratio of 1:1. No significant difference in age was present between these groups (*p* > 0.05). The mean stone size was 8.6 mm (range 1–30). Breakdown by stone location was as follows: upper ureter 13.3%, mid ureter 12.5%, lower ureter 56.6%, renal pelvis 3.5%, upper pole 1.2%, mid pole 1%, lower pole 8.4%, other 3.5%.

**Table 1:** Demographics of patients reported in the studies

Author	Journal	Year	Country	No. of procedures	Sample size (male/female)	Mean age (years) (range)	Mean stone size (range) (mm)
Medium-volume centres							
Schuster [6]	Journal of Urology	2002	USA	27*	25 (13/12)	9.2 (3 to 14)	6 (2–12)
Dogan [7]	BJU International	2004	Turkey	35*	35 (15/20)	6.2 (1 to 14)	8 (4–15)
Satar [8]	Journal of Urology	2004	Turkey	33*	33 (NR)	7.4 (0.75 to 15)	5.3 (3–10)
Al-Busaidy [9]	BJU International	2004	Oman	28*	26 (14/12)	6.5 (2–12)	12.1 (4–22)
Thomas [12]	J Urol	2005	USA	33*	29 (15/14)	7.8 (0.4–12)	6 (3–9)
El-assmy [13]	Journal of Endourology	2006	Egypt	33*	32 (NR)	8.7 (2–15)	7 (4–15)
Ertuhan [16]	Journal of Endourology	2007	Turkey	41*	41 (16/25)	9.5 (3–15)	5.6 (4–10)
Corcoran [17]	J Urol	2008	USA	30*	30	9.7 (2.2–14.4)	8.8 (1.5–25)
Yeow [20]	J Indian Assoc Pediatr Surg	2009	Australia	26*	26 (14/12)	8.2 (0.25–15)	10.3 (3–21)
Chedgy [31]	Urologia Internationalis	2015	UK	32*	21 (13/8)	8.6 (1.4–16)	9.6 (5–20)
Featherstone [33••]	Journal of Paediatric urology	2016	UK	35*	18 (7/11)	10.4 (3.6–15)	13.2 (10–25)
Iqbal [35]	Urology	2016	Pakistan	37*	37 (25/12)	8.37 (NR)	10.01 (NR)
Utangac [36]	JCPSP	2016	Turkey	34*	34 (22/12)	0.8 (0.33–12)	NR
High-volume centres							
Al Busaidy [4]	British Journal of Urology	1997	Oman	50**	43 (29/14)	6.2 (0.5–12)	12.6 (4–22)
Bassiri [5]	Journal of Endourology	2002	Iran	66**	66 (NR)	9 (2–15)	8 (5–15)
Minevich [10]	Journal of Urology	2005	USA	81**	71 (39/32)	7.5 (1–12)	NR
Raza [11]	Journal of Endourology	2005	UK	52**	35 (25/10)	5.9 (0.9–15)	8.8 (3–20)
Gedik [14]	International Urology and Nephrology	2007	Turkey	54**	54 (32/22)	8.5 (1–16)	7.1 (4–12)
Smaldone [15]	Journal of Urology	2007	USA	115**	100 (42/58)	13.2	8.3
Tanaka [18]	Journal of Urology	2008	USA	52**	50 (31/19)	7.9 (1.2–13.6)	8 (1–16)
Kim [19]	Journal of Urology	2008	USA	170**	167 (89/78)	5.2 (0.25–18)	NR
Tanriverdi [21]	Paediatric Surgery International	2010	Turkey	65**	65 (39/26)	9.1 (2–16)	6.1 (3–24)
Turunc [22]	Journal of Endourology	2010	Turkey	66**	61 (NR)	8.1 (0.5–16)	9.5 (3–30)
Ghazaleh [23]	Saudi Journal of Kidney Diseases and Transplantation	2011	Jordan	78**	56 (38/18)	8.2 (6–14)	8.2 (4–20)
Nerli [24]	Journal of Endourology	2011	India	88**	80 (69/11)	9.5 (6–12)	12 (9–15)
Dogan [25•]	Journal of Urology	2011	Turkey	660**	642 (265/377)	7.5 (0.33–17)	10.2 (7–16)
Yucel [26]	World journal of urology	2011	Turkey	54**	48 (28/20)	7.6 (0.75–18)	8.9 (NR)
Atar [27]	Urological research	2012	Turkey	69**	64 (23/41)	4.3 (NR)	NR
Resorlu [28]	Urology	2012	Turkey	95**	95 (53/42)	9.3 (1–17)	14.3 (NR)
Jurkiewicz [29]	Urolithiasis	2013	Germany	157**	126 (66/60)	7.5 (0.8–17)	7.2
Ezkurt [30]	Urolithiasis	2013	Turkey	65**	65 (31/34)	4.3 (0.5–7)	14.66 (7–30)
Sen [32]	Journal of Paediatric urology	2015	Turkey	175**	175 (101/74)	4 (NR)	9.6 (5–20)
Gokce [34]	Urology	2016	Turkey	116**	116 (78/38)	9.5 (NR)	9.4 (NR)
Other							
Guven [37]	Urology	2016^a^	Global (over 23 countries)	192	192 (109/83)	10.3 (NR)	4.56 (1.96–9.43)

^*^Medium volume centre; ^**^High volume centre

^a^Multicentric study

Overall, 2944 procedures were performed with a mean caseload of 87 procedures per study (range 25–660). There were 13 and 20 studies in the medium-volume [6–9, 12, 13, 16, 17, 20, 31, 33••, 35, 36] and high-volume [4, 5, 10, 11, 14, 15, 18, 19, 21–24, 25•, 26–30, 32, 34] groups respectively. Given the paediatric data from the CROES database that was gathered from over 50 centres, it was excluded from this subclassification although the data was used for the overall results [37].

## Outcome Measures

All studies reported SFR, with an overall SFR of 90.4% (range 58–100). Medium-volume centres reported a mean SFR of 94.1% (range 87.5–100). High-volume centres reported a mean SFR of 88.1% (range 58–98.5). Mean number of sessions to achieve stone-free status in medium-volume and high-volume groups was 1.1 and 1.2 procedures/patient respectively (Table 2).

**Table 2:** Results of the studies (stone location, SFR, failure to access and complications)

Author	Stone location (*n*)								SFR (%)	Failures (*n*)	Complications (*n*)
	Upper ureter	Mid ureter	Lower ureter	Renal pelvis	Upper pole	Mid pole	Lower pole	Other stones			
Medium-volume centres											
Schuster [6]	NR	NR	NR	NR	NR	NR	NR	NR	100	0	Stent migration (1), pyelonephritis (1)
Dogan [7]	2	–	33	–	–	–	–	–	97	1	Ureteric perforation (2)
Satar [8]	6	3	26	–	–	–	–	–	94	2	UTI (1)
Al-Busaidy [9]	6	5	17	–	–	–	–	–	92	2	Transient haematuria (4), fever (2)
Thomas [12]	3	5	24	1	–	–	–	–	96	1/33	Extravasation (1)
El-Assmy [13]	2	2	29	–	–	–	–	–	96.9	1/33	Extravasation (1), transient haematuria (1)
Erturhan [16]	4	15	27		–	–	–	–	87.7	5/41	Nil
Corcoran [17]	NR	NR	NR	NR	NR	NR	NR	NR	94	2/30	Ureteral perforation (2), urinoma (1)
Yeow [20]	NR	NR	NR	NR	NR	NR	NR	NR	88.5	3/26	Nil
Chedgy [31]	NR	NR	NR	NR	NR	NR	NR	NR	95	0	UTI (1)
Featherstone [33••]	2	–	5	4	7	3	21	4	89	1	Nil
Iqbal [35]	NR	NR	NR	NR	NR	NR	NR	NR	100	0	Pyelonephritis (2), haematuria (4)
Utangac [36]	4	9	2	–	–	–	–	–	94.1	0	Minimal bleeding (2), ureteral perforation (1), UTI (2)
High-volume centres											
Al Busaidy [4]	9	7	34	–	–	–	–	–	93	3/43	Ureteric perforation (2)
Bassiri [5]	2	5	59	–	–	–	–	–	88	3/66	Transient haematuria (11), pyelonephritis (3), renal colic (1)
Minevich [10]	16	14	28	7	–	–	–	–	98	0	Nil
Raza [11]	0	3	72	–	–	–	–	2	87.2	0	Ureteric perforation (2), urinary retention (1), mild fever (5), mucosal tear (1)
Gedik [14]	3	16	25	–	–	–	–	–	77.8	2	Pyrexia (3)
Smaldone [15]	19	11	37	6	10	–	17	–	91	0	Ureteric perforation/extravasation (5), ureteral stricture (1)
Tanaka [18]	–	–	–	27	–	–	13	11	58	0	Re-admission due to nausea and vomiting (1)
Kim [19]	–	47	19	–	–	–	87	14	98.5	0	Nil
Tanriverdi [21]	5	2	33	–	–	–	–	–	89.2	2	Mucosal lacerations (2), minor haematuria (1)
Turunc [22]	7	9	50	–	–	–	–	–	92.4	5	Pyrexia (1)
Ghazaleh [23]	–	–	–	34	6	4	–	24	94.8	0	UTI (3), haematuria (1)
Nerli [24]	56	–	–	–	–	–	–	24	97.5	2	Intra-operative bleeding (6), self-limiting post-operative bleeding (8), pyrexia (4)
Dogan [25•]	96	73	480	–	–	–	–	21	90	9/660	Stone migration (8), mucosal laceration (1), broken catheter (1), ureteral perforations (5), haematuria (2) (1 intra-op, 1 post-op), post-op pain (2), febrile UTI (20), urinary retention (1), 1 urethral stone (1), late ureterovesical junction obstruction (4)
Yucel [26]	NR	NR	NR	NR	NR	NR	NR	NR	84.3	8/54	Ureteral perforation (3), urosepsis (30), ureteral obstruction with stone fragment (1)
Atar [27]	6	9	54	–	–	–	–	–	85.6	12	Mild haematuria (8), ureteral laceration (8), ureteric perforation (4), urinoma (1), renal colic (5), febrile UTI (9), urinary retention (7), bleeding/false route/perforation intra-operatively (5)
Resorlu [28]	NR	NR	NR	NR	NR	NR	NR	NR	92.6	0	Minor complications (Clavien I/II) 8.4% Major complications (Clavien III–V) nil
Jurkiewicz [29]	NR	NR	NR	NR	NR	NR	NR	NR	98.1	4/15	Ureteral perforation (1), ureterovesical stenosis (1)
Ezkurt [30]	–	–	–	22	10	12	28	–	92.3	5/65	Pyelonephritis (10), haematuria (6), ureteral wall injury (2)
Sen [32]	NR	NR	NR	NR	NR	NR	NR	NR	66	NR	Fever (30), ureteral laceration (8), sepsis (6)
Featherstone [33••]	2	–	5	4	7	3	21	4	89	1	Nil
Gokce [34]	NR	NR	NR	NR	NR	NR	NR	NR	87	3/116	Mucosal injury (8), renal colic (22)
Other											
Guven [37]	NR	NR	NR	NR	NR	NR	NR	NR	89.2	NR	Pain (1), stricture (2)

*NR* not reported

Across all the included studies, the overall complication rate was 11.1% (327/2994). Breakdown by Clavien grade was as follows: Clavien I 69% and Clavien II/III 31% (Table 3). There were no Clavien IV/V complications, and no mortality was recorded across any of the studies.

**Table 3:** Complications reported in studies from medium-volume and high-volume centres

Nature of complication	Clavien grade	Number of complications (*n*)	
		Medium volume	High volume
Post-operative renal colic	I	–	2
Haematuria	I	9	36
UTI/pyelonephritis	I	4	72
Mild fever/pyrexia post-operatively	I	12	34
Urinary retention	I	1	8
Post-operative renal colic	I	–	29
Re-admission due to nausea and vomiting	I	–	1
Urethral stone	I	–	1
Late ureterovesical junction obstruction	III	–	5
Stent migration	III	1	8
Ureteral strictures	III	–	1
Post-operative ureteral stone	III	–	–
Broken catheter	III	–	1
Intra-operative bleeding/false passage/ureteral perforation/tear/laceration/submucosal wire	III	10	63
Stone migration	III	–	8
Total	–	37	269

Medium-volume and high-volume studies had overall complication rates of 6.9% (37/530) and 12.1% (287/2222) respectively. There was no significant difference in major or minor complications between these two groups. The overall failure rate was 2.5% (76/2944). Most of them were due to failure to access the paediatric ureter.

## Discussion

### Findings and Implications of Our Review

This is the largest review on paediatric URS to date and reveals an overall SFR of 90.4% and an overall complication rate of 11.1%. Over two thirds of these complications were Clavien I. Importantly, there was no significant difference in SFR or complication rates between medium-volume and high-volume centres.

### PCNL in Paediatric Population

Percutaneous nephrolithomy (PCNL) and shockwave lithotripsy (SWL) represent the key alternative interventions to URS. Whilst the former can achieve high stone-free rates in a single procedure and is not limited by failure to access the ureter such as can occur in URS, it carries a worse morbidity profile, notably in the form of haemorrhagic complications. Bhageria et al. reported transfusion rate of 9% in their retrospective cohort of 95 children undergoing PCNL [38]. Miniaturisation of standard equipment (< 24Fr) has delivered a key strategy for improving its safety status both in adult and paediatric populations. Multiple studies have confirmed higher incidence of haematuria and renal extravasation associated with the use of larger tract sizes [39]. PCNL can now even be delivered in the ‘micro’ format using a 4.5Fr tract with final SFRs reported between 80 and 100% [39]. Its use for treatment of ureteric stones however remains less valuable [40].

### SWL in Paediatric Population

Shockwave wave lithotripsy is a minimally invasive option, with a relatively short learning curve and generally minor complications [40]. It has traditionally been the first-line intervention for paediatric stone disease. However, it can necessitate multiple sittings and in children generally requires administration of general anaesthetic. Additionally, SFRs are less predictable with stone recurrences commonly due to incomplete stone clearances [41].

### Future Trends in Ureteroscopy

With increased uptake of URS, it looks set to reach an increasing number of endourological milestones. URS has also undergone the miniaturisation process. Utangac et al. recently reported using a micro-ureteroscope (4.5Fr along entire length) in 11 children with a median stone size of 10.5 mm [41]. Stone-free status was achieved in all cases. There were no intra-operative complications and only one case of transient haematuria post-operatively. This novel modification may prove extremely valuable and allow better ureteric cannulation/navigation with fewer cases of access failure. However, further studies are needed comparing it with standard URS.

## Limitations of Our Study

Whilst this study represents the largest review to date on paediatric URS, there are certain limitations, which the authors acknowledge. Results have been included from predominantly retrospective studies with age ranges spanning development of the urinary tract from infancy to adult state. The heterogeneity of available evidence did not allow for formal meta-analysis to be performed. In comparison, we did find a relatively higher stone-free rate with lower complications in medium-volume centres. However, we feel that this might reflect higher complexity of cases in established endourology high-volume centres. Similarly, training and guidance on ‘tips and tricks’ of ureteroscopy might help improve outcomes in less well-established paediatric stone centres [42, 43].

## Conclusion

URS is a safe and effective treatment for the treatment of stone disease among the paediatric population. Medium-volume centres can achieve equally high SFRs and safety profiles as high-volume centres. The findings of this review may therefore support increased uptake of URS in centres performing fewer procedures each year.
